# High-Intensity GPS-Derived Parameters in Semi-Professional Soccer: Home vs. Away Roles of the Team and Their Relationship with the Match’s Final Score

**DOI:** 10.3390/s24154891

**Published:** 2024-07-27

**Authors:** Jorge Carlos-Vivas, Juan Manuel Franco-García, David Manuel Mendoza-Muñoz, Santiago Gómez-Pomares, Jorge Pérez-Gómez

**Affiliations:** 1 Physical Activity for Education, Performance and Health (PAEPH) Research Group, Faculty of Sport Sciences, University of Extremadura, 10003 Cáceres, Spain; jorgecv@unex.es; 2 Health, Economy, Motricity and Education (HEME) Research Group, Faculty of Sport Sciences, University of Extremadura, 10003 Cáceres, Spain; jmfrancog@unex.es; 3 Faculty of Sport Sciences, University of Extremadura, 10003 Cáceres, Spain; dmendozaj@alumnos.unex.es (D.M.M.-M.); sgomez@alaves.com (S.G.-P.)

**Keywords:** monitoring, performance, soccer, team sports, training

## Abstract

High-intensity activities are related to success in football. We looked at whether high-intensity activity differed between home and away matches and its impact on the final score. Thirty football players (20.3 ± 0.8 years) were recruited from a Spanish semi-professional team. Footballers wore a GPS device to monitor high-intensity parameters from competition matches. The final score of every match was also recorded. Playing at home showed greater total distance (TD) > 24 km/h, >27 km/h, >85% Vmax, and the number of sprints > 24 km/h (all *p* < 0.05) than playing away. Positive correlations were also found between the match score and high-speed running (HSR) distance covered by the team per minute (r = 0.401), TD > 21 km/h (r = 0.417), TD > 24 km/h (r = 0.343), number of sprints > 24 km/h (r = 0.337), and HSR per minute (r = 0.459) (all *p* < 0.05). The results suggest that playing at home is associated with greater high-intensity effort than playing away. Moreover, the volume of high-intensity effort influences the final score.

## 1. Introduction

Soccer is a cooperative-opposition sport requiring prolonged aerobic involvement, alternating with unpredictable and intermittent high-intensity actions such as accelerations, decelerations, changes in direction, sprints, jumps, and charges [1,2], with players making frequent intensity changes in their multidirectional movements and actions, from short high-intensity efforts to long periods of low and moderate intensity [2,3]. In this context, the players’ performances are influenced by various tactical, technical, biomechanical, and physiological demands [2,4], Achieving sporting results in matches and throughout the season requires optimal levels of physical, tactical, technical, and mental capacity [5].

Players cover, during a professional match, a total distance of 9–14 km [6,7]. The total distance covered is divided into different intensities, with the largest percentage of this distance being covered at low intensity (<14 km/h), around 12% at high-intensity (14–21 km/h), and approximately 4% at very high-intensity runs (21.1–24 km/h) and sprints (>24 km/h) [3,7]. Moreover, high-intensity actions are the ones that determine the performance and team success [8]. Recently, global positioning system (GPS) measurement technology has been used to obtain this type of data, providing complete and valuable information on different parameters in training sessions and competition matches [9,10]. This information helps coaches and physical trainers to manage and quantify their players’ efforts, prioritise their decisions to maximise each player’s performance, minimise fatigue, and reduce the risk of any type of injury [11,12]. Hence, planning and periodisation of training of training have benefited from the use of this technology, which attempts to replicate the intensities and movement patterns that occur in matches during the training sessions [13,14], and adapting the loads according to the physical demands of each playing position [3,15].

Previous research has demonstrated the influence of various situational variables on player performance during matches [16,17,18]. In football, for both physical and technical-tactical actions, the influence of situational variables on players at the behavioural level has been shown [19]. Thus, the impact of the location of the match (playing at home or away) [16,20], the period of the match, and the level of the opposing team (high-, medium-, or low-level teams) on the performance of players during the matches have been demonstrated [20,21]. In addition to the physical performance of the players, the number of points needed to avoid relegation also has an impact, as the pressure to get further away from the relegation zone seems to increase the total distance travelled by players during matches [20].

During a competitive season, peak periods for high-intensity running and sprinting can vary by up to 38% and 75%, respectively [22]. As previously mentioned, contextual variables are influential for the performance of players and the physical and technical demands of matches, such as the team’s style of play and the sociocultural aspects of the league [23]. Playing against teams considered stronger by the players themselves also results in higher career values [24,25]. The present research aims to analyse the influence of playing at home or as a visitor for semi-professional players of Spanish football on the high-intensity parameters derived from the GPS. Similarly, Aquino et al. (2020) analysed different contextual variables in professional Brazilian players [24], obtaining that the players who played at home presented higher values in terms of total distance and high-intensity running.

Some studies have shown that semi-professional players travel lower overall distances and distances at higher speeds during matches compared to professional players, which is generally associated with the lower technical demands of lower leagues [26,27]. Due to the fact that, in earlier times, budgetary constraints reduced access to sports technology, there is little evidence on high-intensity parameters in Spanish semi-professional soccer and the influence that different conditioning factors, such as the final score or playing home matches, may have. In this regard, there are increasing advances in accessibility by professional teams to microelectromechanical systems [28], and having baseline data on these parameters could be influential in the control of training loads and overall season planning to optimise team performance.

Furthermore, as mentioned, this study seeks to explore the associations between different high-intensity GPS parameters and match results. Gonçalves et al. (2021) observed that the distance per minute travelled at high intensity was higher in matches won than in matches drawn and lost by Brazilian professional players [29].

Therefore, the present study aimed (1) to describe and compare the GPS-derived high-intensity parameters between the home and away roles of a semi-professional football team during the full season, and (2) to explore the associations between the GPS-derived high-intensity parameters and the final match score. Thus, we hypothesise that (1) high-intensity workload parameters will be higher at home matches than away matches, and (2) a positive relationship will be found between high-intensity workload parameters and the result of the match.

## 2. Materials and Methods

### 2.1. Design

A cohort study was conducted with a team at the third tier of the Spanish league during the 2021–2022 season. The external workload was monitored daily for each training session and competition. However, only the GPS-derived high-intensity parameters from the competition matches were considered in this study, based on its first aim. The final score of each competition match was also recorded, considering the second aim of the study.

### 2.2. Subjects

Convenience sampling was performed. Thirty football players (age: 20.3  ±  0.8 years; height: 184.4  ±  6.6 cm; weight: 75.1  ±  6.8 kg) were recruited from a Spanish semi-professional team in the third tier to participate in this study. The Bioethics and Biosafety Committee at the University of Extremadura approved the study procedures (reference number: 97/2023) in accordance with the Helsinki Declaration on Human Research statements and guidelines. Since the data reported in this retrospective study were collected as part of the players’ routine data monitoring in industrial practice, informed consent was not deemed necessary [30]. However, participants were informed that their data would be used for academic-scientific purposes, as well as for the control and monitoring of their training and performance.

### 2.3. Procedures

Data were collected during the 2021–2022 season from a semi-professional Spanish football team. A total of 44 weeks, including 38 competition days, were monitored. However, only data from competition days were computed in this study, considering their nature and aims. To obtain valid and reliable information, every football player wore a GPS device (Vector S7/G7, Catapult Sports, Melbourne, Australia) positioned between their scapulae into a customised, tight-fitting neoprene garment [31] as part of their daily monitoring routines within all training and competition sessions. Each player wore the same unit throughout the season. These devices were taken outside and activated 15–30 min beforehand to ensure suitable GPS signal quality [32]. No problems were detected in the GPS devices, nor in the quality of the signals that could affect the accuracy of the data collected, during the course of the study.

According to the manufacturer’s manual, this system includes the following specifications: (1) dual indoor and outdoor tracking capability; (2) global positioning, 10 Hz GPS, GLONASS, and SBAS (or 18 Hz GPS); (3) local positioning, 10 Hz Catapult ClearSky; (4) Communication, Ultra-Wideband (UWB), and Bluetooth 5; (5) Heart rate, ECG derived (Vector S7, G7), and Polar Gymlink Compatible; (6) Accelerometer, 3D +/− 16G, sampled at 1 kHz, Provided at 100 Hz; (7) gyroscope, 2000°/s at 100 Hz; (8) Magnetometer, D ± 4900 μT at 100 Hz; (9) Six hours battery life; (10) Advanced inertial analysis and sport-specific algorithms; football-specific goalkeeping analysis (G7).

### 2.4. Measures

The following protocol was used to ensure accurate data collection using GPS: Before starting the player participation in the training or competition sessions, the GPS unit was positioned in the tight-fitting neoprene garment, and the activation of the turn-on light was checked before the start of the session. At the end of the workout, the GPS unit was removed, and it directly entered the docking station to store information. Data were stored using updated specialised Catapult software (Sprint 5.1.7, Catapult Sports, Melbourne, Australia). Considering the aims of the present study, only GPS-derived high-intensity variables of the stored external load parameters were considered. Specifically, the parameters used were as follows: (1) total distance covered over 14 km/h (TD > 14 km/h); (2) high-speed running distance covered (HSR), defined as the total distance covered over 21 km/h; (3) total distance covered between 21 and 24 km/h (TD > 21 km/h); (4) total distance covered between 24 and 27 km/h (TD > 24 km/h); (5) total distance covered over 27 km/h (TD > 27 km/h); (6) total distance covered over 85% of individual maximum velocity (TD > 85% Vmax); (7) high-speed running distance covered per minute (HSR per min); (8) the total number of sprints over 24 km/h (No Sprints > 24 km/h); and (9) the number of runs over 85% of maximum velocity (No 85% Vmax). The speed thresholds were selected according to the GPS manufacturer’s default guidelines. For computations and analyses, the sum of the external workload data registrations of all players who took part in each competitive match was calculated and considered.

Additionally, the final score of every competition match was recorded to explore the possible associations between GPS-derived high-intensity parameters and the final score of the match.

### 2.5. Statistical Analysis

The Statistical Package for the Social Sciences Statistical SPSS (version 25.0; IBM SPSS Inc., Armonk, NY, USA) was used for statistical procedures and computations. Data are presented as mean and standard deviation (SD). For the comparative analysis between the team’s home and away matches, the sum of the external loads of all players who participated in each match was considered for each of the dependent variables analysed.

Data were checked for normality and homogeneity using Shapiro–Wilk and Levene’s tests, respectively. Subsequently, inferential tests were conducted. An independent samples *t*-test was performed to analyse the between-team role (home vs. away) differences in all dependent variables. Moreover, the Hedge’s g effect size with a 95% confidence interval was calculated. The effect size thresholds were interpreted as follows [33]: ≤0.2, trivial; >0.2, small; >0.6, moderate; >1.2, large; >2.0, very large; >4.0, nearly perfect. Additionally, Pearson’s correlation coefficients were calculated to explore the possible associations between different GPS-derived high-intensity parameters and the final match score. The correlation thresholds were defined as follows [33]: ≤0.1, trivial; >0.1 to ≤0.3, small; >0.3 to ≤0.5, moderate; >0.5 to ≤0.7, large; >0.7 to ≤0.9, very large; and ≥0.9, nearly perfect. Differences were considered significant at *p* ≤ 0.05.

## 3. Results

Table 1 compares the different high-intensity GPS-derived parameters between the team’s home and away roles during the season. The results revealed significantly greater TD over 24 km/h (*p* = 0.035; *g* = 0.63), TD over 27 km/h (*p* = 0.021; *g* = 0.76), TD over 85% Vmax (*p* = 0.039; *g* = 0.68), and the number of sprints over 24 km/h (*p* = 0.014; *g* = 0.82) for the home role than for the away role matches of the team. A trend toward higher HSR (*p* = 0.055; *g* = 0.63) and the number of runs at velocities over 85% Vmax (*p* = 0.052; *g* = 0.64) was also observed in favour of the home role. However, no differences were found for TD over 14 km/h, TD over 21 km/h, and HSR per min (*p* > 0.05).

Table 2 displays the associations between the different high-intensity GPS-derived parameters and the match scores. The main outcomes showed significant positive correlations between the match score and HSR (*r* = 0.401; *p* = 0.013), TD > 21 km/h (*r* = 0.417; *p* = 0.009), TD > 24 km/h (*r* = 0.343; *p* = 0.035), number of sprints > 24 km/h (*r* = 0.337; *p* = 0.039), and HSR per minute (*r* = 0.459; *p* = 0.004). However, non-significant associations were found between the match score and TD over 27 km/h, TD over 85% Vmax, number of runs at velocities over 85% Vmax, and TD over 14 km/h (*p* > 0.05).

## 4. Discussion

This study aimed to describe and compare GPS-derived high-intensity parameters between home and away matches of a semi-professional football team over a full season, and to explore the associations between GPS-derived high-intensity parameters and final match outcome. Depending on the location of the match, the results showed that in home matches, the TD covered above 24 and 27 km/h, the TD covered above 85% Vmax, and the number of sprints above 24 km/h were significantly higher than in away matches. In addition, a positive correlation was found between the match result and the high-intensity GPS parameters. Thus, our initial hypothesis was partially fulfilled. A wide variety of factors can be attributed to these findings, such as motivation, fan support, weather conditions, tactical advantage, and travel fatigue [34,35,36] involved in playing at home.

Consistent with our results, research by Aquino et al. (2020) examined the physical performance of football players during home and away matches [24]. Their results showed that players from home teams exhibited greater total distance covered at higher speeds and more sprints compared to players from away teams. Furthermore, these findings confirm the results obtained in a meta-analysis showing a home-field advantage in football [37]. Many decades ago, some studies showed the advantage of playing at home compared to playing away [38]. Pollard indicated that teams playing at home won around 75% more points than teams playing away [38]. Crowd support is a relevant factor that helps with playing at home. A recent study found that without spectators during the COVID-19 pandemic, home advantage was reduced and teams showed lower performance [39]. More studies agreed that local support is an important factor positively influencing players who play at home [40,41,42]. Bilasic et al. detected a decrease in home advantage (nearly 50%) when there is a reduction in fans at the stadium [40]. Isin and Gomez Ruano observed that home teams have benefit over away teams with and without spectators [41]. Vandoni et al. detected home advantages in empty stadiums [42], so factors other than local crowd are also involved in home advantage. It is known that motivation is relevant in the context of fatigue induced by exercise [43]. Barte et al. saw that soccer players experimentally motivated were able to increase their performance compared to the control group without motivation [43]. The increase in performance of soccer players playing at home could be related to fan support. This could be one of the reasons why, in the current study, greater high-intensity performance was shown when playing at home.

In home matches, higher stress has been observed in soccer players, measured by cortisol levels, at post games compared to away matches [44]. On the other hand, game tactics seem to be more varied and structured, with higher complexity and different patterns at home than away in soccer matches [45]. These aspects could indicate that playing at home makes players feel the necessity to win. As a consequence, they increase high-intensity actions, as observed in the present study. In addition to game location determining tactical and technical performance in soccer matches, many actions like shots, goals, attacking movements, crosses, and dribbles are higher playing at home than away [46], and many of these aspects require high-intensity actions, supporting the results obtained in this study.

In addition, the positive correlation found between high-intensity parameters and match results coincides with those found in several studies [16,17,47]. The results showed that teams with better physical performance in terms of distance covered at high speed and number of sprints had a higher probability of winning the match. Other findings also showed positive associations between physical performance and match outcomes, showing that teams that had a higher number of sprints and a greater distance covered at high speed were more likely to have a positive match outcome [46]. However, it is important to keep in mind that results may vary according to the context and specific characteristics of each team and match (tactical strategy, technical quality of the players, teamwork, among others) [19,48,49]. Home advantage in team sports has an important role in determining the outcome of a game and for allowing a differentiation between home and visiting teams [46]. This reason can explain why this study found that the volume of high-intensity efforts influences the final score. Although there is some evidence of positive associations between high-intensity parameters and the final match result, these are not the only determining factors, and an integrated approach is required to fully understand football performance.

The main limitation of this study is the sample size, as only one team from a specific category was analysed, and it may be difficult to extrapolate the results to other teams or categories and result in low external validity. In future studies on GPS-derived high-intensity parameters between home and away matches in semi-professional football teams, the same procedure should be repeated for more teams and different league categories. This will allow us to observe the trend in our results with other similar studies. In addition, it may be interesting to study the variables treated in our research, considering positional tactics and team pressure blocks (low, medium, or high), depending on team formation and the system of play. Moreover, it may be of interest to analyse the workloads of microcycles in which the team assumes the role of the host in competitions, as opposed to microcycles in which it performs as an away team.

## 5. Conclusions

The present study’s outcomes suggest that teams playing at home exert greater high-intensity efforts compared to when they play away. Moreover, there is an influence of the volume of high-intensity efforts on the final score of the match, as a positive and direct association was observed between some GPS-derived high-intensity parameters and team success on competition days.

## 6. Practical Applications

This research has significant practical applications in the fields of coaching and strategic team planning. Teams can use these results to adjust their training strategies according to their role in the match (home or away). On the one hand, if teams are shown to exert more effort and cover more total distance at high intensities in home matches compared to away matches, they should focus their training on improving endurance and the ability to perform high-intensity efforts in these specific contexts. Therefore, training sessions should be selected and designed with the specific efforts that the team will be performing in the next match in mind, just as coaches consider other characteristics of the opponent for this process. On the other hand, away teams can focus on strategies to maintain optimal energy levels and performance during the match, despite not being in their usual environment.

The results indicate that there is a positive and direct relationship between high-intensity parameters and team success on match days. This suggests that the amount of high-intensity effort may be a critical factor in determining the outcome of a match. Coaches can use this information to strategically plan key moments in a match when high-intensity effort is required to maximise the chances of success. They can also identify patterns and trends in team performance based on high-intensity parameters, allowing them to adjust their tactics and match strategies.

Our findings suggest that teams playing at home exert greater high-intensity effort. This has important implications for performance management and recovery. Teams can implement specific recovery strategies to minimise the negative effects of high-intensity efforts during home matches. These may include active recovery techniques, training periodisation, workload, and dietary adjustments.

In conclusion, this study highlights the need for coaches and trainers to choose the most appropriate playing strategy depending on the venue of the next football match, as well as planning different tasks to be carried out during weekly training in order to satisfy the specific behavioural patterns required for the next match, depending on whether it is at home or away. Of course, the multifactorial perspective that determines the effort a team puts in during the competition must always be considered, and is not only dependent on the role of home or away, but is an additional factor to be considered.

## Figures and Tables

**Table 1:** Comparison of different high-intensity GPS-derived parameters between home and away roles of the team during the season.

	Team Role		Mean Difference (95% CI)	*p*	Hedges’ *g* (95% CI)
	Home (N = 19)	Away (N = 19)			
**TD > 14 km/h** (m)	22,939.53 (4034.72)	21,314.84 (2180.35)	1624.68 (−509.14 to 3758.52)	0.131	0.49 (−0.15 to 1.14)
**HSR** (m)	5047.00 (929.32)	4566.21 (503.72)	480.79 (−11.03 to 972.61)	0.055 ^†^	0.63 (−0.02 to 1.28)
**TD > 21 km/h** (m)	2965.21 (388.13)	2813.68 (288.00)	151.53 (−73.34 to 376.40)	0.180	0.43 (−0.21 to 1.08)
**TD > 24 km/h** (m)	2081.84 (577.37)	1752.58 (312.70)	329.26 (23.75 to 634.77)	0.035 *	0.69 (0.04 to 1.35)
**TD > 27 km/h** (m)	789.32 (358.10)	570.16 (170.62)	219.16 (34.60 to 403.72)	0.021 *	0.76 (0.11 to 1.42)
**TD > 85% Vmax** (m)	442.47 (288.03)	288.74 (122.73)	153.74 (8.06 to 299.40)	0.039 *	0.68 (0.03 to 1.33)
**HSR per min** (m·min^−1^)	64.53 (11.00)	61.68 (11.42)	2.84 (−4.53 to 10.23)	0.440	0.25 (−0.39 to 0.89)
**Nº Sprints > 24 km/h** (number)	200.58 (41.69)	171.16 (26.99)	29.42 (6.31 to 52.53)	0.014 *	0.82 (0.16 to 1.48)
**Nº 85% Vmax** (number)	30.74 (13.40)	23.37 (8.66)	7.37 (−0.05 to 14.79)	0.052 ^†^	0.64 (−0.01 to 1.29)

Abbreviations: TD > 14 km/h, total distance covered by the team over 14 km/h; HSR, high-speed running distance covered by the team; TD > 21 km/h, total distance covered by the team between 21 and 24 km/h; TD > 24 km/h, total distance covered by the team between 24 and 27 km/h; TD > 27 km/h, total distance covered by the team over 27 km/h; TD > 85% Vmax, total distance covered by the team over the 85% of maximum velocity; HSR per min, high-speed running distance covered by the team per minute; Nº Sprints > 24 km/h, the total number of sprints over 24 km/h; Nº 85% Vmax, number of running over the 85% of maximum velocity; *p*, *p*-value at alpha level 0.05; Hedges’ g (95% CI), Hedges’s g effect size magnitude with 95% confidence interval. * Significant between-group differences at *p* ≤ 0.05. ^†^ Between-group trend differences with *p* > 0.5 to 0.7.

**Table 2:** Associations between the different high-intensity GPS-derived parameters and match scores.

	Final Match Score	
	*r*	*p*
**TD > 14 km/h**	0.235	0.155
**HSR**	0.401	0.013 *
**TD > 21 km/h**	0.417	0.009 *
**TD > 24 km/h**	0.343	0.035 *
**TD > 27 km/h**	0.243	0.142
**TD > 85% Vmax**	0.212	0.201
**HSR per min**	0.459	0.004 *
**Nº Sprints > 24 km/h**	0.337	0.039 *
**Nº 85% Vmax**	0.222	0.181

Abbreviations: TD > 14 km/h, total distance covered by the team over 14 km/h; HSR, high-speed running distance covered by the team; TD > 21 km/h, total distance covered by the team between 21 and 24 km/h; TD > 24 km/h, total distance covered by the team between 24 and 27 km/h; TD > 27 km/h, total distance covered by the team over 27 km/h; TD > 85% Vmax, total distance covered by the team over the 85% of maximum velocity; HSR per min, high-speed running distance covered by the team per minute; Nº Sprints > 24 km/h, the total number of sprints over 24 km/h; Nº 85% Vmax, number of running over the 85% of maximum velocity; *p*, *p*-value at alpha level 0.05. * Significant correlation at *p* ≤ 0.05.

## Data Availability

The datasets used during the current study are available from the corresponding author upon reasonable request.
